# Potential of Endophytic Bacterium *Paenibacillus* sp. PHE-3 Isolated from *Plantago asiatica* L. for Reduction of PAH Contamination in Plant Tissues

**DOI:** 10.3390/ijerph13070633

**Published:** 2016-06-24

**Authors:** Xuezhu Zhu, Li Jin, Kai Sun, Shuang Li, Wanting Ling, Xuelin Li

**Affiliations:** Institute of Organic Contaminant Control and Soil Remediation, College of Resource and Environmental Sciences, Nanjing Agricultural University, Nanjing 210095, China; zhuxuezhu@njau.edu.cn (X.Z.); jinlinj0411@126.com (L.J.); 2013203015@njau.edu.cn (K.S.); 2015103062@njau.edu.cn (S.L.); lixuel@njau.edu.cn (X.L.)

**Keywords:** phenanthrene, PAHs, biodegradation, endophytic bacterium, co-metabolism, catechol 2,3-dioxygenase

## Abstract

Endophytes are ubiquitous in plants, and they may have a natural capacity to biodegrade polycyclic aromatic hydrocarbons (PAHs). In our study, a phenanthrene-degrading endophytic *Paenibacillus* sp. PHE-3 was isolated from *P. asiatica* L. grown in a PAH-contaminated site. The effects of environmental variables on phenanthrene biodegradation by strain PHE-3 were studied, and the ability of strain PHE-3 to use high molecular weight PAH (HMW-PAH) as a sole carbon source was also evaluated. Our results indicated that pH value of 4.0–8.0, temperature of 30 °C–42 °C, initial phenanthrene concentration less than 100 mg·L^−1^, and some additional nutrients are favorable for the biodegradation of phenanthrene by strain PHE-3. The maximum biodegradation efficiency of phenanthrene was achieved at 99.9% after 84 h cultivation with additional glutamate. Moreover, the phenanthrene biodegradation by strain PHE-3 was positively correlated with the catechol 2,3-dioxygenase activity (ρ = 0.981, *p* < 0.05), suggesting that strain PHE-3 had the capability of degrading HMW-PAHs. In the presence of other 2-, 3-ringed PAHs, strain PHE-3 effectively degraded HMW-PAHs through co-metabolism. The results of this study are beneficial in that the re-colonization potential and PAH degradation performance of endophytic *Paenibacillus* sp. PHE-3 may be applied towards reducing PAH contamination in plants.

## 1. Introduction

With increasing anthropogenic activities, polycyclic aromatic hydrocarbons (PAHs) pose substantial concerns owing to their widely known potential toxicity. PAHs containing more than three aromatic rings have been referred to as high molecular weight (HMW) PAHs in the environmental microbiology literature. PAHs containing four fused benzene rings are classed as recalcitrant, and not easily being degraded. Due to their elevated octanol-water partition coefficients (*K*_ow_), HMW-PAHs may separate into organic phases, soil and sediment organic matter, and membranes of living organisms [1]. An abundance of HMW-PAHs was found in vegetables from contamination sites [2]. Biomagnifications of HMW-PAHs through trophic transfer to food webs pose potential risks to human and ecological health.

Biodegradation of HMW-PAHs by microorganisms is one of the main ways to remove them from the environment. Various microbial and fungal groups have been found to be capable of utilizing PAHs—including naphthalene, phenanthrene, and pyrene—as sole carbon sources, or to co-metabolize PAHs [3,4,5]. Specific exogenous microorganisms—such as bacteria and fungi, which have better degrading capabilities for a wider range of aromatic compounds—are usually introduced into the soil to enhance the degradation rate. Furthermore, the rhizosphere is the interface between plant roots and soil where interactions among a myriad of microorganisms and invertebrates affecting biogeochemical cycling, plant growth, and tolerance to biotic and abiotic stress [6]. Moreover, bacteria that are found in plant species but do not visibly harm host plants are called endophytic bacteria. Philippot et al. (2013) suggested that plants can transmit specific microorganisms from one generation to another, and several endophytes were able to exit the root interior to colonize the rhizosphere [6]. Berg et al. (2005) also reported that these endophytic bacteria often belong to specific genera commonly found in soil [7]. Several PAH-degrading endophytic bacteria, which could degrade PAHs in plant tissues, have been isolated from plants grown in PAH-contaminated soils [8,9]. 

All the plants have the possibility to be the host plants, and specific endophytic bacteria colonizing internal tissues of host plants could effectively stimulate certain transcription levels of specific genes [10]. Soleimani et al. (2010) reported that inoculated plants created higher levels of water-soluble dehydrogenase activity, which is an important enzyme in the biodegradation of PAHs [11]. Plants can provide co-substrates for bacteria to enhance biodegradation of HMW-PAHs [12]. Thus, we hypothesized that highly PAH-degrading endophytic bacteria can be applied in order to reduce the accumulation of PAHs in plant tissues. As such, inoculating plants with PAH-degrading endophytic bacterial strains would represent a potential manner in which to decrease PAH contamination in plants.

For this purpose, more PAH-degrading endophytic bacterial strains need to be isolated. The objectives of this study were to isolate PAH-degrading endophytic bacteria from PAH-contaminated plants and to evaluate their capabilities of degrading PAHs in vitro, which will be a benefit for exploring the re-colonization potential and PAH degradation performance of endophytic bacteria in a target plant. The results will provide a new perspective in the reduction of plant PAH contamination risks in PAH-contaminated sites by inoculating plants with endophytic bacteria.

## 2. Materials and Methods

### 2.1. Isolation of PAH-Degrading Endophytic Bacteria

Healthy plants (*P. asiatica* L., *Setaria viridis* (L.) *Beauv.*, and *Avena fatua* L.) were collected from PAH-contaminated sites near Sinopec Yangzi Petrochemical Co., Ltd. (Nanjing, China) Each plant sample was preserved at 4 °C until further use within seven days. Luria-Bertani (LB) medium was used for the enrichment of PAH-degrading bacteria. Mineral salt (MS) medium was used as the basal medium for isolating PAH-degrading endophytic bacteria and evaluating the capabilities of these microbes for degrading PAHs. Phenanthrene was used for the isolation of endophytic bacteria as a representative of PAHs. Stock solutions of individual and PAH mixtures were prepared in methanol and used in all degradation experiments.

Plant tissues were sterilized after being immersed in 75% (*v*/*v*) ethanol-water solution for 3–5 min and immersed in 0.1% (*v*/*v*) mercuric chloride solution for 3–5 min. Subsequently, these plant tissues were washed thrice with sterile deionized water (18.2 MΩ-cm) to remove the surface sterilizing agents and were cultivated on an LB plate at 30 °C for confirmation that all external bacteria were eliminated [13]. After successfully having their surface disinfected, the plant tissues were aseptically ground.

The dilution was incubated in flasks containing 100 mL of MS media supplemented with 50 mg·L^−1^ of phenanthrene. The aliquots were transferred weekly to fresh MS medium supplemented with 50 mg·L^−1^ of phenanthrene at least four times prior to the isolation of the bacterial strains. All flasks were incubated in the dark on a rotary shaker at 30 °C and 150 r·min^−1^. Isolation and purification procedures were performed on MS medium agar plates coated on the surface with a layer of 50 mg·L^−1^ phenanthrene, and subsequently incubated at 30 °C. The size and color of the isolated colonies were recorded. The bacterial strains were selected based on colony morphology and color.

### 2.2. Identification of PAH-Degrading Endophytic Bacteria

The strain was classified based on 16S rRNA gene sequence analysis and its physiological and biochemical characters. The biochemical characters of strain PHE-3 were tested according to the methods described by Dong and Cai (2001) [14]. 16S rRNA gene fragments from the isolated strains were prepared following the method described by Byers et al. (1998) [15]; genomic DNA was used as a template to amplify the extracted 16S rRNA gene fragments through PCR using the universal primers 16S-27F (5′-AGAGTTTGATCCTGGCTCAG-3′) and 16S-1492R (5′-TACCTTGTTACGACTT-3′) (Invitrogen Co., Ltd., Shanghai, China). The amplification reactions were performed on a DNA Engine Thermal Cycler (PTC-200, BIO-RAD, Foster City, CA, USA). The 25-μL PCR mixture contained 1-μL template, 2.5 μL of 10 × Taq DNA polymerase buffer, 5 mmol·L^−1^ MgCl_2_, 1-μL dNTPs at 2.5 mmol·L^−1^, 3.75 pmol each of the forward and reverse primers, and 0.5 mL of 2.5 units Taq polymerase. Sequencing was performed at the Nanjing Genscript Biotechnology Company, Limited (Nanjing, China). The 16S rRNA gene sequences were queried against the GenBank database [16] and the microgenetic analysis was performed using the Clustalx 1.83 and MEGA 6.0 programs.

### 2.3. Biodegradation of PAHs by Endophytic Bacterium

Ten phenanthrene-degrading endophytic bacterial strains, which could use phenanthrene as the sole source of carbon and energy, were isolated through a selective enrichment culture procedure. Among them, strain PHE-3 and strain PHE-5 could degrade more than 90% phenanthrene in the medium within seven days. Since strain PHE-5 (*Stenotrophomonas maltophilia* sp.) was identified as a pathogenic bacterium, strain PHE-3 isolated from (*P. asiatica* L.) was selected for further investigation. The cells were used as inocula in degradation studies after reaching the stationary phase through suspension in fresh MS medium at an optical density *OD*_600 nm_ of 1.0 (10^8^ cells·mL^−1^). The degradation of PAHs was monitored in 50-mL flasks containing 20 mL of MS medium containing PAHs as the sole carbon sources, and 1-mL aliquots of the strain suspension were added to the prepared flasks. The control flasks were inoculated with sterilized MS medium to assess the impact of abiotic factors on the PAHs stability. All cultures were incubated on a rotary shaker (150 r·min^−1^) at 30 °C.

#### 2.3.1. Capacity of Degrading Phenanthrene

To measure the degradation rates of phenanthrene by strain PHE-3, strains were cultivated in MS medium supplemented with 50 mg·L^−1^ phenanthrene as the sole carbon source for 96 h. Triplicate flasks were retrieved every 12 h for detection of PAH levels, using degradation kinetics equations to represent the biodegradation ability.

To assess the effects of the initial phenanthrene levels on the biodegradation of phenanthrene, strains were cultivated in the MS media supplemented with phenanthrene at 25, 50, 100, 200, and 300 mg·L^−1^, respectively. After 96 h cultivation, triplicate flasks from each treatment were retrieved for detection of phenanthrene levels.

The temperature experiments included temperatures of 20, 30, 37, and 42 °C. In the pH experiments, the initial pH value of the MS medium was adjusted to 4.0, 5.0, 6.0, 7.0, 8.0, 9.0, and 10.0. After 96 h incubation, triplicate flasks from each treatment were retrieved for the detection of phenanthrene residues.

#### 2.3.2. Activities of Catechol 2,3-dioxygenase

The strains were cultivated in the MS media supplemented with phenanthrene at 25, 50, 100, 200, and 300 mg·L^−1^. After 84 h cultivation, quadruplicate flasks from each treatment were retrieved for the detection of catechol 2,3-dioxygenase (C_2,3_O) activities. The culture broth was harvested and centrifuged at 8000 r·min^−1^ for 5 min at 4 °C to collect cells. For determination of C_2,3_O activity, the cell paste was resuspended in 10 mmol·L^−1^ phosphate buffer (pH 7.5) and disrupted with an ultrasonic oscillator at 200 W in an ice bath for 30 min. Particulate matter was removed from the extract by centrifugation at 12,000 r·min^−1^ for 30 min at 4 °C. The supernatant was used for the assay of C_2,3_O activity, which was according to published spectrophotometric procedure [17]. The reaction mixture (total 3.0 mL) contained 2.2 mL phosphate buffer, 0.4 mL 20 μmol·L^−1^ catechol, and 0.4 mL cell lysates. The increase of absorbance due to the formation of 2-hydroxy-muconic semialdehyde was measured at 375 mm. One unit of its specific activity is defined as the increase of 0.001 *OD*·min^−1^.

#### 2.3.3. Capabilities of Degrading Other PAHs

##### Capabilities of Degrading Other PAHs Separately

One 2-ringed PAH (naphthalene), two 3-ringed PAHs (fluorene and phenanthrene), two 4-ringed PAHs (fluoranthene and pyrene), and one 5-ringed PAH (Benzo[a]pyrene) were selected as representatives for PAHs to investigate the capability of strain PHE-3 to biodegrade PAHs. The strains were cultivated in MS medium supplemented with PAH separately. The initial concentration of each PAH in the medium was as follows: 500 mg·L^−1^ naphthalene, 100 mg·L^−1^ fluorene, 100 mg·L^−1^ phenanthrene, 100 mg·L^−1^ fluoranthene, 50 mg·L^−1^ pyrene, and 10 mg L^−1^ benzo[a]pyrene. After 7 d cultivation, triplicate flasks from each treatment were retrieved for detection of PAH levels.

##### Capacities of Degrading a Mixture of PAHs

The aliquots were suspended in the MS medium supplemented with a mixture of six PAHs, including 500 mg·L^−1^ naphthalene, 100 mg·L^−1^ fluorene, 50 mg·L^−1^ phenanthrene, 50 mg·L^−1^ fluoranthene, 50 mg·L^−1^ pyrene, and 10 mg·L^−1^ benzo[a]pyrene. After 7 d cultivation, triplicate flasks from each treatment were retrieved for detection of PAH levels.

### 2.4. Effects of Additional Nutrients on Phenanthrene Degradation

Glucose, sucrose, citric acid, and yeast were selected as the representatives for carbon nutrients, and NH_4_NO_3_, (NH_4_)_2_SO_4_, glutamate, and tryptone were selected as the representatives for nitrogen nutrients. The aliquots were suspended in the MS medium supplemented with 100 mg·L^−1^ phenanthrene and each of the additional nutrients at 50 mg·L^−1^. After 84 h cultivation, triplicate flasks from each treatment were retrieved for detection of PAH levels.

### 2.5. Detection of PAH Residues by HPLC

The PAHs were extracted from the MS media with methanol, which was added to the medium at the ratio of 7:3 (*v*/*v*), ultrasonically extracted for 30 min, and centrifuged at 12,000 r·min^−1^ for 10 min, followed by filtration through 0.22-µm filters.

The PAH levels in the prepared samples were quantified using a high-performance liquid chromatography (HPLC; LC-20AT; Shimadzu, Kyoto, Japan) equipped with a 4.6 × 150-mm reverse-phase C_18_ column using methanol-water (90:10) as the mobile phase at a flow rate of 0.8 mL·min^−1^. Chromatography was performed at 40 °C using a detection wavelength of 245 nm.

### 2.6. Statistical Analyses

The statistical significance of any differences between treatments was subjected to one-way analysis of variance (ANOVA). Differences with *p* values < 0.05 were considered statistically significant. The kinetics equations for PAH degradation by strain PHE-3 were calculated using regression analyses.

## 3. Results and Discussion

### 3.1. Isolation and Identification of Strain PHE-3

**Figure 1:**
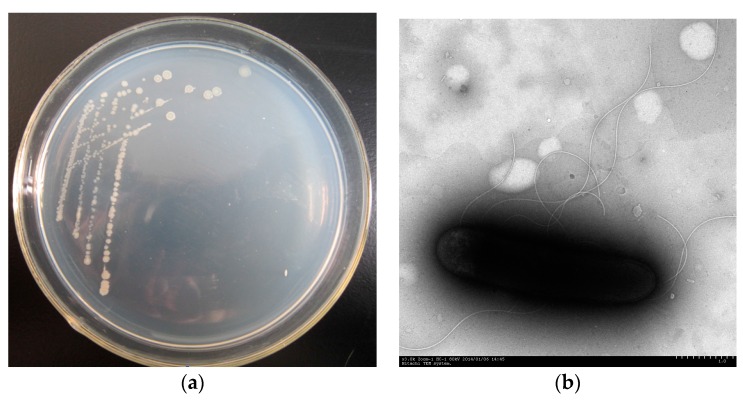
Photograph of (**a**) colonies of strain PHE3 on a Luria-Bertani (LB) medium plate and (**b**) electron micrograph of strain PHE-3 (×3.0 K).

The endophytic strain PHE-3 was isolated from *P. asiatica* L. grown in a PAH-contaminated soil, which could utilize phenanthrene (up to 300 mg·L^−1^) as the sole source of carbon and energy. The colonies and a cell micrograph of strain PHE-3 are shown in Figure 1. The physiological and biochemical characteristics are presented in Table 1. The colonies of strain PHE-3 showed faint yellow color, and were small, round, semitransparent, and convex with a glistening surface and an irregular shape with a slightly serrated border (Figure 1a). The cells of strain PHE-3 were long, aerobic, with peritrichous and a large number of extracellular polysaccharide, and gram-positive rods (Figure 1b). Strain PHE-3 could hydrolyze starch, although the results for the other reactions were negative (Table 1).

**Figure 2:**
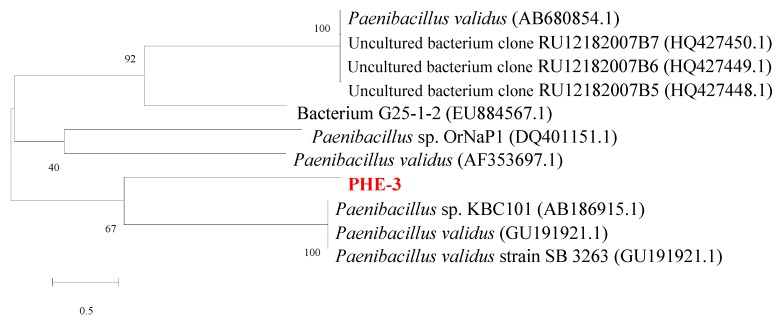
Phylogenetic tree of 16S rRNA gene sequence for strain PHE-3 and related bacteria.

Based on BLAST sequence comparison, the 16S rRNA of strain PHE-3 was 99.0% similar to that of *Paenibacillus* sp. (Figure 2). According to its 16S rRNA, and its physiological and biochemical characters, strain PHE-3 could be considered a *Paenibacillus* sp. strain, a genus which has typically been found to have the capability of promoting plant growth [18]. Previous research suggested that *Paenibacillus* stains had great potential for degrading various persistent organic pollutants (POPs), including kraft lignin [19], dibenzothiophene (DBT) and alkyl DBTs [20], hexenuronic acid [21], and PAHs [22]. Some bacteria belonging to the *Paenibacillus* genus that were isolated from the petroleum-contaminated sediment and salt marsh rhizosphere could use naphthalene or phenanthrene as a sole carbon source [23]. Moreover, several *Paenibacillus* sp. strains were found to have the capability of both using PAHs and tolerating higher concentrations of arsenic, cadmium, and lead [24]. Furthermore, *Paenibacillus* strains were usually found to be able to increment plant growth [25], and produce a novel biosurfactant that was capable of desorbing PAHs from soil and assisting with the removal of PAHs [26]. 

### 3.2. Biodegradation of Phenanthrene by Strain PHE-3

**Figure 3:**
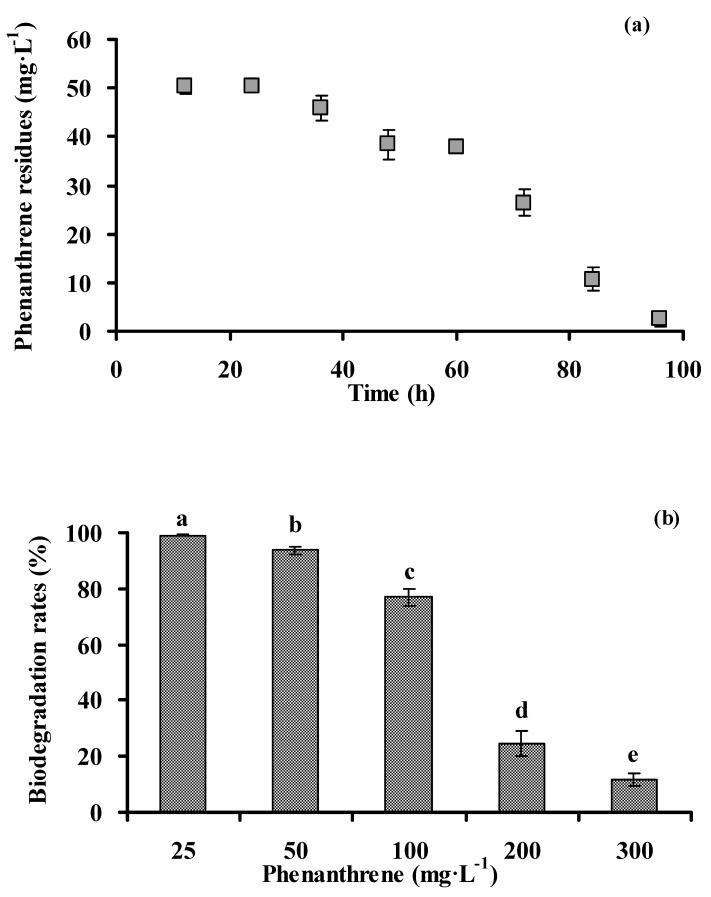
Biodegradation of phenanthrene by strain PHE-3 at different cultivation times (**a**) and under different initial concentrations (**b**) at 30 °C for 96 h. Different lowercase letters indicate significant differences among treatments (*p* < 0.05).

As shown in Figure 3a, phenanthrene was rapidly degraded after 24 h, and the degradation rates were low during the first 24 h. The residual of phenanthrene and the cultivation time had a significant negative correlation (ρ = −0.954, *p* < 0.01). The degradation kinetics of phenanthrene (50 mg·L^−1^) was shown as an equation. From this equation, the half-life (*T*_1/2_) value of phenanthrene was 23 h, thereby suggesting that phenanthrene could be quickly degraded by strain PHE-3.

*C*_phenanthrene_ = 123.73 × *е*^‒0.0302 t^ (by strain PHE-3, r = 0.7135) (1)

where *C*_phenanthrene_ represents the residual concentrations of phenanthrene in the medium (mg·L^−1^), and t represents the incubation time (h).

Strain PHE-3 could effectively degrade phenanthrene at 50, 100, 150, 200, 250, and 300 mg·L^−1^, while the degradation rates significantly decreased with the increasing phenanthrene levels (ρ = −0.978, *p* < 0.01) (Figure 3b). When the levels of phenanthrene were less than 100 mg·L^−1^, the degradation rates of phenanthrene were more than 77% after 4 d inoculation.

### 3.3. Catechol 2,3-dioxygenase Activity

**Figure 4:**
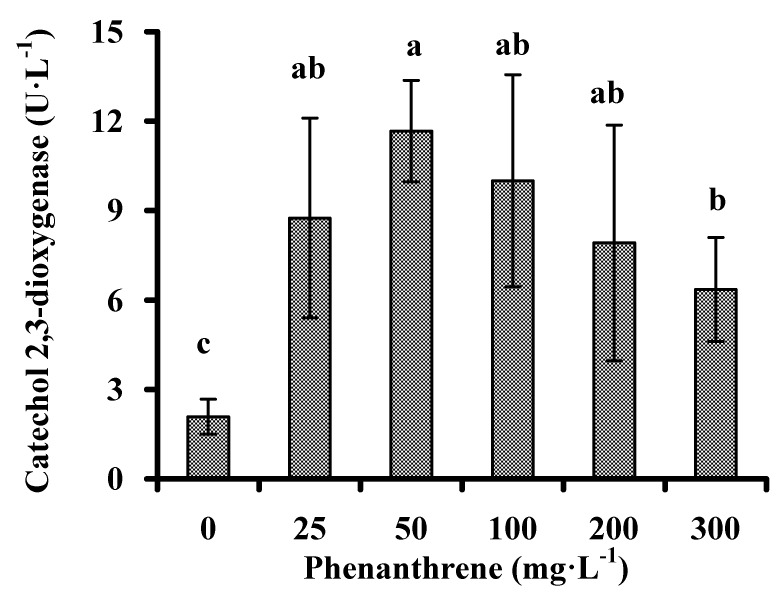
Catechol 2,3-dioxygenase activities of strain PHE-3 cultivated in the medium with different levels of phenanthrene at 30 °C after 84 h. Different lowercase letters indicate significant differences among treatments (*p* < 0.05).

C_2,3_O is a member of the super family of extradiol dioxygenases, and catalyzes the ring cleavage of catechol and substituted catechols. It is also an important mechanism to degrade PAHs by bacteria [27]. Given the activities of C_2,3_O with respect to the utilization of low molecular weight PAHs (LMW-PAHs) and HMW-PAHs [28], C_2,3_O activities were detected in order to evaluate the capability of strain PHE-3 to degrade PAHs. As shown in Figure 4, phenanthrene promoted C_2,3_O activity, and the biodegradation of phenanthrene was positively correlated with the C_2,3_O activity (ρ = 0.981, *p* < 0.05). The C_2,3_O level showed a peak in the medium with 50 mg·L^−1^ phenanthrene, while the C_2,3_O activities decreased with increasing phenanthrene levels when phenanthrene levels were more than 50 mg·L^−1^ (ρ = 0.990, *p* < 0.05), thereby suggesting that reduction of the C_2,3_O activities might be the reason why the biodegradation of phenanthrene occurred less in the media with high levels of phenanthrene (200, 250, and 300 mg·L^−1^). Based on its promotion of C_2,3_O activities, strain PHE-3 might have the capability to degrade HMW-PAHs.

### 3.4. Biodegradation of Other PAHs

**Figure 5:**
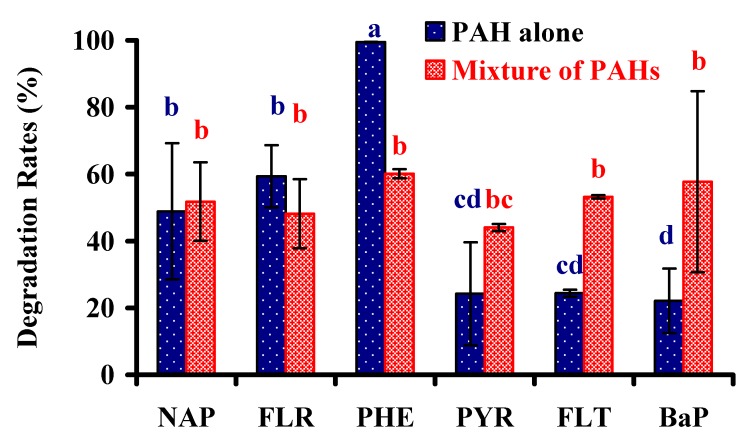
Biodegradation of polycyclic aromatic hydrocarbons (PAHs) by PHE-3 cultivation in the medium with a single PAH or a mixture of PAHs at 30 °C after 7 d. Different lowercase letters indicate significant differences among treatments (*p* < 0.05). Note: Naphthalene (NAP), Fluorene (FLR), Phenanthrene (PHE), Pyrene (PYR), Fluoranthene (FLT), and Benzo[a]pyrene (BaP).

After being cultivated in a medium containing either a individual PAH or a mixture of six PAHs, strain PHE-3 was able to simultaneously degrade naphthalene, fluorene, phenanthrene, fluorene, pyrene, and benzo[a]pyrene (Figure 5). High efficiency was observed for naphthalene degradation in strain PHE-3, which was similar to another *Paenibacillus* strain reported by Pepi (2009) which degraded 87% naphthalene from media after 20 h incubation [24]. Moreover, the degradation of PAHs occurred rapidly, with 53.2% of the fluorene, 44.0% of the pyrene, and 57.7% of the benzo[a]pyrene having degraded after seven days by inoculating strain PHE-3 in the medium containing a mixture of PAHs; however, only 24.4% of the fluorene, 24.2% of the pyrene, and 22.1% of the benzo[a]pyrene were degraded in the absence of other PAHs during the same period.

These results suggested that naphthalene, fluorine, and phenanthrene were supplied as co-substrates; the degradation of fluorene, pyrene, and benzo[a]pyrene proceeded at a relatively rapid rate compared to the rate which degradation occurred when the substrates were alone. *Paenibacillus* sp. PHE-3 could biodegrade PAHs through co-metabolism, as it was found by Thavamani et al. (2012) that *Paenibacillus* sp. PHE-3 could use phenanthrene as a co-substrate for degradation of benzo[a]pyrene [29]. Since HMW-PAHs are quite tolerant to microbial attack, until now, the reported degradation rate of benzo[a]pyrene by microorganisms was generally quite modest. Due to its co-metabolism, strain PHE-3 would have great potential for bioremediation of HMW-PAHs. However, the biodegradation of phenanthrene was inhibited in presence of HMW-PAHs. Zhong et al. reported that pyrene and fluoranthene could inhibit the production of 1-hydroxy-2-naphthoic acid to decrease the biodegradation of phenanthrene (2010) [30]. For improving its biodegradation of phenanthrene, the optimal conditions were evaluated in the following study.

### 3.5. Optimal Environmental Conditions for Biodegradation of Phenanthrene

#### 3.5.1. pH and Temperature

**Figure 6:**
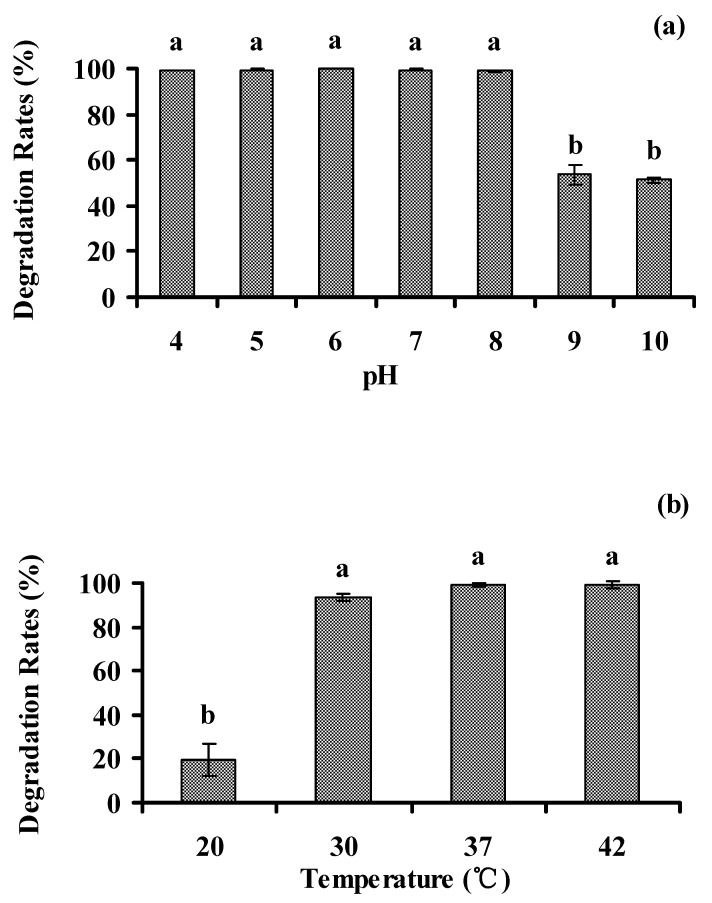
Effects of pH (**a**) and temperature (**b**) on the degradation of phenanthrene by PHE-3. Bacteria were grown in a mineral salt (MS) medium supplemented with 50 mg·L^−1^ phenanthrene, and phenanthrene levels in the culture supernatant were detected after 7 d of incubation (30 °C in Figure 6a; pH 7.0 in Figure 6b). Different lowercase letters indicate significant differences among treatments (*p* < 0.05).

Several bacteria were reported to effectively biodegrade PAHs in the medium with initial pH from 4.0 to 10.0 [31,32]. To test optimal pH of the medium for strain PHE-3, the degradation tests were performed at initial pH from 4.0 to 10.0, and temperatures from 20 to 42 °C (Figure 6a). Similar to other bacteria reported by Fu and Lin, strain PHE-3 could effectively biodegrade phenanthrene (more than 99%) when the initial pH of the media were between 4.0–8.0, and an initial pH value of 8.0 was key in the degradation of phenanthrene [31,32]. The degradation of phenanthrene decreased with increasing pH values when the pH of the medium was more than 8.0 (ρ = –0.795, *p* < 0.05). Meanwhile, the biodegradation rates of phenanthrene by strain PHE-3 were more than 93% when the cultivation temperature were from 30 to 42 °C, while the biodegradation rate was 19% at 20 °C (Figure 6b). These results show that the optimal conditions for strain PHE-3 to degrade phenanthrene were as follows: pH between 4.0–8.0 and temperature between 30 °C–42 °C, thereby suggesting that strain PHE-3 could perform well under high temperature and alkalescent condition. Ferguson et al. (2008) also found that a *Paenibacillus* strain was capable of rapidly mineralizing hydrocarbons at 42 °C in an Antarctic hydrocarbon-contaminated site [33].

#### 3.5.2. Additional Carbon and Nitrogen Nutrients

The degradation of phenanthrene could be enhanced by adding additional carbon and nitrogen nutrients (Table 2). The degradation rates of phenanthrene were all more than 95% after 84 h cultivation with additional nutrients (*p* < 0.05). When glucose, sucrose, citric acid, and yeast were separatedly added in the medium, the degradation rates of phenanthrene were increased by 22.2%, 24.7%, 26.3% and 26.3%, respectively (*p* < 0.05). In addition, the degradation was also significantly enhanced by nitrogen nutrients including NH_4_NO_3_, (NH_4_)_2_SO_4_, glutamate, and tryptone (Table 2). Additional glutamate enhanced phenanthrene degradation by 22.5%–26.78% and achieved maximal degradation rates (99.9%) in our study (*p* < 0.05). These results suggest that strain PHE-3 has some potential of co-metabolism, which is supported by previous research that found that LMW-organic compounds could enhance the biodegradation of HMW-PAHs via co-metabolism [34,35]. Likewise, this is the same as for other bacteria, whose degradation of PAHs could be enhanced by adding nitrogen nutrients [36]. Since these beneficial organic compounds and nitrogen nutrients (including glucose, sucrose, citric acid, NH_4_NO_3_, (NH_4_)_2_SO_4_, glutamate, and tryptone) can be found in plant tissues, strain PHE-3 would degrade PAHs well in plant tissues.

### 3.6. Possible Application for PAH-Degrading Endophytic Bacteria

It has been reported that endophytic *Paenibacillus* strains could be inoculated in plants and promote plant growth. The plant growth-promoting activities of endophytic *Paenibacillus* strains included N_2_-fixing [37], forming biofilm to display greater biocontrol, and increasing chlorophyll content in plants [18]. In our study, the endophytic bacterial strain *Paenibacillus* sp. PHE-3 could effectively degrade PAHs in vitro, indicating it could be beneficial for re-colonizing a target plant for reducing the risk of plant PAH contamination.

## 4. Conclusions

Endophytes with the capacity to highly degrade PAHs in vitro may have significant implications for re-colonizing a target plant and reducing PAHs residue in vivo. In our study, endophytic *Paenibacillus* sp. PHE-3, which could effectively degrade naphthalene, fluorene, phenanthrene, fluoranthene, pyrene, and benzo[a]pyrene both separately and together, was isolated from *P. asiatica* L. that was grown in PAH-contaminated soil. The biodegradation of PAHs could be enhanced by some co-substrates including LMW-PAHs, suggesting that strain PHE-3 might perform well in PAH-contaminated plants. Our results will provide a novel endophytic bacterium for bioremediation of PAH-contaminated sites and plants.

## Figures and Tables

**Table 1:** Physiological and biochemical characters of strain PHE-3.

Reaction	Results	Reaction	Results
Glucose fermentation test	−	Nitrate reaction	−
Methyl red staining	−	Voges-Proskauer reaction	−
Starch hydrolysis	+	Hydrogen sulfide test	−
Indole production	−	Gelatin liquefaction testing	−
Citrate utilization test	−	Phenylalanine deaminase	−

Note: +, positive reaction; −, negative reaction.

**Table 2:** Effects of additional nutrients on the degradation of phenanthrene. Bacteria were grown in MS medium supplemented with 100 mg·L^−1^ phenanthrene and each of the other additional nutrients. Phenanthrene levels in the culture supernatant were detected after 84 h of incubation (at 30 °C, pH 7.0).

Carbon Sources	Degradation Rate (%)	Nitrogen Sources	Degradation Rate (%)
CK	73.12 ± 3.03 d	CK	73.12 ± 3.03 d
Glucose	95.31 ± 1.05 c	NH_4_NO_3_	99.77 ± 0.13 a
Sucrose	97.80 ± 1.02 b	(NH_4_)_2_SO_4_	99.28 ± 0.38 a
Citric acid	99.41 ± 0.40 a	Glutamate	99.90 ± 0.03 a
Yeast	99.45 ± 0.44 a	Tryptone	95.62 ± 0.30 c

Note: Different lowercase letters indicate significant differences among treatments (*p* < 0.05).
